# Addressing Differentiation in Live Human Keratinocytes by Assessment of Membrane Packing Order

**DOI:** 10.3389/fcell.2020.573230

**Published:** 2020-10-21

**Authors:** Danuta Gutowska-Owsiak, Ewa I. Podobas, Christian Eggeling, Graham S. Ogg, Jorge Bernardino de la Serna

**Affiliations:** ^1^University of Gdansk, Intercollegiate Faculty of Biotechnology of University of Gdansk and Medical University of Gdansk, Gdansk, Poland; ^2^Medical Research Council Human Immunology Unit, National Institute for Health Research Oxford Biomedical Research Centre, Medical Research Council Weatherall Institute of Molecular Medicine, University of Oxford, Oxford, United Kingdom; ^3^Institute of Biochemistry and Biophysics, Polish Academy of Sciences, Warsaw, Poland; ^4^Faculty of Biology, Institute of Genetics and Biotechnology, University of Warsaw, Warsaw, Poland; ^5^Institute of Applied Optics and Biophysics, Friedrich-Schiller-University Jena, Jena, Germany; ^6^Leibniz Institute of Photonic Technologies e.V., Jena, Germany; ^7^Faculty of Medicine, National Heart and Lung Institute, Imperial College London, London, United Kingdom

**Keywords:** keratinocyte differentiation, cornification, membrane heterogeneity, spectral imaging, high throughput, membrane stiffness, Laurdan fluorescence

## Abstract

Differentiation of keratinocytes is critical for epidermal stratification and formation of a protective *stratum corneum*. It involves a series of complex processes leading through gradual changes in characteristics and functions of keratinocytes up to their programmed cell death via cornification. The *stratum corneum* is a relatively impermeable barrier, comprised of dead cell remnants (corneocytes) embedded in lipid matrix. Corneocyte membranes are comprised of specialized lipids linked to late differentiation proteins, contributing to the formation of a stiff and mechanically strengthened layer. To date, the assessment of the progression of keratinocyte differentiation is only possible through determination of specific differentiation markers, e.g., by using proteomics-based approaches. Unfortunately, this requires fixation or cell lysis, and currently there is no robust methodology available to study keratinocyte differentiation in living cells in real-time. Here, we explore new live-cell based approaches for screening differentiation advancement in keratinocytes, in a “calcium switch” model. We employ a polarity-sensitive dye, Laurdan, and Laurdan general polarization function (GP) as a reporter of the degree of membrane lateral packing order or condensation, as an adequate marker of differentiation. We show that the assay is straightforward and can be conducted either on a single cell level using confocal spectral imaging or on the ensemble level using a fluorescence plate reader. Such systematic quantification may become useful for understanding mechanisms of keratinocyte differentiation, such as the role of membrane in homogeneities in stiffness, and for future therapeutic development.

## Introduction

The epidermis, in the form of a stratified and desquamating tissue is a fascinating feature of many organisms; it retains very distinct characteristics which enable it to exert function, shielding us from external threats and preventing fluid evaporation. An understanding is emerging on how these unique features contribute to the important protective role of the epidermis; these lessons are learned both from a physiological setting and through the perspective of skin diseases. For example, keratinocyte differentiation and cornification are essential for prevention of allergic diathesis by forming a functional skin barrier (Kubo et al., 2012; Flohr et al., 2014; Horimukai et al., 2014; Venkataraman et al., 2014). Cornification encompasses a series of processes leading to the programmed keratinocyte death, which results in formation of a cellular remnant known as “corneocyte.” Corneocytes, embedded in a lipid-enriched matrix (Eckhart et al., 2013) provide mechanical hardness, while the lipids support elastic properties of the *stratum corneum*. In addition, at the cellular level, the lamellar organization of lipids and their lateral packing properties prevent evaporation (Iwai et al., 2012; Janssens et al., 2012; Narangifard et al., 2018), forming a seal over the moisture trapped in the *stratum corneum*. This phenomenon is achieved by high abundance of hydrophilic compounds, known as Natural Moisturizing Factor (NMF).

**Figure 1:**
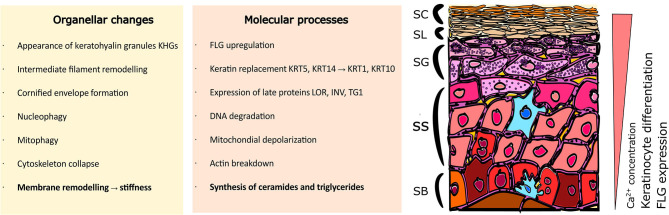
Organellar and molecular changes in keratinocytes during the differentiation process. Stratified epidermis consists of multiple layers of epidermal keratinocytes characterized by increasing advancement of cell differentiation. Cells undergo extensive organellar and molecular alterations as they gradually move to the upper layers. Where SC, Stratum Corneum; SL, Stratum Lucidum; SG, Stratum Granulosum; SS, Stratum Spinosum; SB, Stratum Basale; FLG, filaggrin; KRT5, keratin 5; KRT14, keratin 14; KRT1, keratin 1; KRT10, keratin 10; LOR, loricrin; INV, involucrin; TG1, transglutaminase 1.

Multiple processes take place during the cornification advancement in the epidermis; these occur simultaneously both at the organellar and molecular levels (Figure 1). The characteristics of progression in keratinocyte differentiation *in vitro* are reflected by multiple morphological changes (i.e., related to the cell shape and adherence) and distinct organellar transformations). For instance, the appearance and maturation of keratohyalin granules (KHGs) (Gutowska-Owsiak et al., 2018), nuclear condensation and extrusion (Gdula et al., 2013; Rogerson et al., 2018), cytoskeleton collapse (Gutowska-Owsiak et al., 2018), and alterations in mitochondria (Ipponjima et al., 2020); in some of those events, autophagy has been shown to be involved (Akinduro et al., 2016). At the molecular level, shifts in cytokeratin expression have been documented (keratin 5, keratin 14 are progressively replaced with keratin 1 and keratin 10) while additional proteins are upregulated, e.g., involucrin and transglutaminase as well as the markers of late differentiation, defined by the expression of late envelope proteins (e.g., involucrin, filaggrin, loricrin) (Marshall et al., 2001; Candi et al., 2005, 2016; Sandilands et al., 2009; Eckhart et al., 2013). These differentiation-dependent proteins contribute to barrier strengthening by incorporation into the insoluble cornified envelope (CE; also known as cornified cell envelope, CCE) or by supporting the hydration of the stratum corneum (Kezic et al., 2009).

Along with the expression of protein markers and organellar changes, an overall increase in cell stiffness can be observed (Plasencia et al., 2007; Janssens et al., 2012; Wennberg et al., 2018). This stiffness is a consequence of two main processes; the formation of a rigid, protein-based CE and extensive lipid remodeling leading to specialized lipid profile adaptation (Ponec et al., 1987; Schmidt et al., 1991; López et al., 2007). Specifically, the high phospholipid and cholesterol content is gradually replaced by a predominance of ceramides and saturated triglycerides (Ponec et al., 1988), especially in conditions where stratification is supported. These lipid composition variations lead to more ordered membrane packing (Janssens et al., 2012), higher stiffness and consequently, changes in the relative lateral heterogeneity properties of the plasma membrane (Bernardino de la Serna et al., 2016). This selection has specific functional properties; the ceramides and free fatty acids provide attachment points for the CE proteins (Behne et al., 2000; López et al., 2007; Akiyama, 2017) and a scaffold to replace the plasma membrane by a corneocyte lipid envelope (CLE), which links CE with the lipids of the intercellular matrix (Elias et al., 2014). It is believed that ceramide enrichment displaces the balance between ordered and disordered regions to support membrane heterogeneity, where higher order regions work as docking molecular anchors supporting the CLE function and ultimately, stabilizing the CE structure. Regulation of ceramide synthesis is mediated via peroxisome proliferator-activated receptor (PPAR) pathways (Chon et al., 2015) associated with keratinocyte differentiation (Mao-Qiang et al., 2004); disturbances in these pathways result in clinical manifestations (Akiyama, 2017).

To date, no robust experimental readout for the assessment of differentiation advancement in live primary keratinocytes in real-time is available. Protein-targeted detection methods, such as the expression quantification by proteomics-based approaches, as well as other commonly used, e.g., immunostaining or western blot, require cell permeabilization or lysis and are only suitable for end-point measurements.

Previous studies have revealed the gel nature of the membranes in the stratum corneum (Plasencia et al., 2007). Here, we built upon the known lipid profile variation and ceramide enrichment during keratinocyte differentiation; we hypothesized that lipid lateral packing and order increase can be used as a hallmark for quantitative assessment of live-cell cornification advancement. For this purpose, we took advantage of the lipophilic polarity-sensitive membrane dye Laurdan, purposely designed by Weber and Farris (1979) to encompass an electron-donor and electron-acceptor, which displays large solvent-dependent fluorescence shifts. Laurdan ubiquitously distributes at the plane of the plasma membrane, regardless of its lipid lateral packing properties, and is oriented parallel with the phospholipid's hydrophobic tails in membranes. Moreover, its emission spectra and location are independent of phospholipid head groups and notably, indirectly report about the relative packing order by sensing the degree of water penetration in its vicinity (Parasassi et al., 1990, 1994, 1997; Bagatolli et al., 1998, 1999); the studies showed that Laurdan could sense the well-known phase transition from fluid to gel at 41C in in 1,2-dipalmitoyl-sn-glycero-3-phosphocholine (DPPC) bilayers. Most of these environmentally-sensitive probes display an increase in charge separation when excited in polar solvents resulting in a larger dipolar moment (Lakowicz, 1999); the penetration of water molecules into a bilayer formed of loosely packed lipids allow more rotational and translational freedom to the probe, yielding an emission shift toward longer wavelengths. On the contrary, the probe emission shifts toward the blue when its motion is more restricted due to sensing a *milieu* with lesser water content. Ratiometrically measuring the emission intensities at 440 and 490 nm renders the Laurdan “general polarization index” (GP), which allows relative quantification of the membrane order or degree of lipid lateral packing (Parasassi et al., 1990, 1997; Sezgin et al., 2015). This method has been used in visualizing native membrane microdomains in planar supported bilayers, giant unilamellar vesicles (de la Serna et al., 2009; Bernardino de la Serna et al., 2013) and in living cells (Gaus et al., 2003; Owen et al., 2011; Carugo et al., 2017; Gutowska-Owsiak et al., 2018; Bernabé-Rubio et al., 2019).

Even though the membrane order can only be measured indirectly, and being aware that the stiffness of a membrane influences can be in principle characterized by fluorescence polarization or fluorescence anisotropy, in this paper we introduced membrane lateral packing order as a readout for keratinocyte differentiation advancement. Using single-cell based confocal spectral imaging or a fluorescence plate reader for high-throughput screening of cellular ensembles we utilize the Laurdan general polarization index assessment of membrane order as an efficient readout for determination of the progression of keratinocyte differentiation in living cells.

## Materials and Methods

### Human Keratinocyte Culture and Calcium Switch

Normal human epidermal keratinocytes (NHEKs) were purchased from Lonza (Lonza, Basel, Switzerland, neonatal, pooled) and cultured in keratinocyte KBM-2 media (Lonza, Basel, Switzerland) at conditions supporting proliferation ([Ca2+] = 0.06 mM) and subcultured by accutase treatment (Sigma Aldrich, Dorset, United Kingdom). To promote terminal differentiation, calcium switch was performed by adjusting calcium level do the final concentration of [Ca2+] = 1.5 mM for a period of 24 h before immunofluorescent labeling. To adjust to the desired calcium concentration a calcium switch was conducted over a period of 24 h by replacing the culture media with media adjusted to the desired calcium concentration by adding CaCl2 (Sigma Aldrich, Dorset, United Kingdom sigma).

### Recombinant Filaggrin

The expression plasmid was constructed using a SLIC method. Briefly, the nucleotide sequence encoding 7th FLG domain with N-terminal 6-His-tagged SUMO protein sequence was cloned into pET28 vector using BamHI i XhoI restrictions sites. The construct was transformed into *E. coli* BL21-CodonPlus-RIL and propagated in LB liquid media with antibiotics, followed by autoinduction media (Formedium AIM- Super Broth) for 48 h at 18°C. The cells were sonicated in lysis buffer (10 mM Tris pH 8, 150 mM NaCl, 10 mM imidazole) with protease inhibitors cocktail. The purification procedure was performed on an ÄKTA™Xpress chromatography system. Lysate was loaded on Ni-NTA Agarose column (Qiagen). On-column cleavage by SUMO protease (4 μg/1 ml) was carried out in elution buffer (10 mM Tris pH 8, 150 mM NaCl, 300 mM imidazole) for 8 h at 10°C. The protein was purified by desalting and on Ni-NTA Agarose columns. Flow though fractions were loaded into a Superdex 200 column (GE Healthcare) and analyzed by SDS-PAGE; protein identification was carried out by MALDI-TOF MS.

### Fluorescent Antibody Staining and Confocal Microscopy Imaging

NHEKs were grown and calcium-switched in eight-well cell culture slides (Beckton Dickinson), fixed and permeabilized by neat acetone. Blocking was carried out in freshly made blocking buffer (5% FCS, Sigma-Aldrich, Gillingham, Dorset, United Kingdom; 2% BSA, Sigma-Aldrich, Gillingham, Dorset, United Kingdom in PBS). For the primary antibody anti-filaggrin antibody (mouse monoclonal 15C10 from Leica Biosystems, Milton Keynes, United Kingdom) and anti-keratin 14 (clone EPR1612 from GeneTex, Irvine, CA, United States) were used; specificity of the antibody was verified prior with both recombinant human FLG and on epidermal lysates (Supplementary Figure 1). The staining was followed by secondary antibody labeling (anti-mouse Alexa 488 and anti-rabitt Alexa 568 Life Technologies/ThermoFisher Scientific, Waltham, MA, United States), all carried out in PBS. To visualize nuclei NucBlue reagent (Hoechst, Life Technologies/ThermoFisher Scientific, Waltham, MA, United States) was used. The cover-slides were mounted with Mowiol-488 (Sigma-Aldrich, Gillingham, Dorset, United Kingdom).

Imaging acquisition was carried out on a Zeiss 780 inverted confocal microscope (Zeiss, Jena, Germany), by recording 2D images or 3D z-stacks. We used 488 nm excitation line and detected from 500–550nm. Images were postprocessed using Zen Software (Zeiss, Jena, Germany) and ImageJ National Institutes of Health, Bethesda, MD, United States).

### Immunocytochemistry

Cells were grown in culture slides (BD Biosciences, San Jose, CA, United States) until 80% confluent, and were subjected to calcium switch for 24 h. The staining was conducted with anti-E-cadherin antibody (Biolegend, San Diego, CA, United States). An EnVision+ DAB polymer immunohistochemistry system (Dako) was used for both for visualization.

### Intracellular Western Blot

Intracellular filaggrin and keratin-14 detection was carried out on cells cultured on 96 well plates that were used for the high throughput Laurdan screen, following data acquisition on the plate reader. Briefly, the cells were washed in PBS and fixed in 3.7% formaldehyde; 0.1% Triton X-100 was used for permeabilization and 2% BSA was used for blocking. Anti-filaggrin clone FLG01 (Abcam, Cambridge, United Kingdom) was verified with rhFLG and on epidermal lysate (by WB) as above. Anti-filaggrin FLG01 and anti-keratin 14 (clone EPR1612 from GeneTex, Irvine, California, United States) were diluted in Odyssey blocking buffer (Lincoln, NE, United States) and incubated for 2 h. After extensive wash (0.1% Tween 20) secondary antibodies (IRDye goat anti-mouse 800 and goat anti-rabbit 680; Li-Cor, Lincoln, NE, United States) were used for 1 h incubation. The data were acquired on Li-Cor Odyssey Scanner and analyzed in Image Studio software and statistical significance was determined by one-way ANOVA test with Tukey's multiple comparison.

### Quantification of Membrane Order by Spectral Imaging

Spectral imaging of the different membrane samples was performed on a Zeiss LSM 780 confocal microscope equipped with a 32-channel GaAsP detector array. Excitation laser at 405 nm was used and the lambda detection range was set between 415 and 691 nm, and intervals set at 8.9 nm for the individual detection channels. This permitted the coverage of the whole spectrum with the 32 detection channels. The images were saved in .lsm file format and analyzed with a custom plug-in compatible with Fiji/ImageJ, as described later. Selection of regions of interest was done using ImageJ and the quantification of the GP index from these regions was done as explained below.

### Quantification of Plasma Membrane Order in Plate Reader

Measurements of the plasma membrane stiffness were carried out on live NHEKs monolayers, employing either a fluorometer (CLARIOstar, BMG LABTECH, Ortenberg, Germany) or optical microscopy using spectral imaging on a Zeiss 780 inverted microscope. We used an environmental polarity sensitive probe, 6-dodecanoyl-2-dimethylamino naphthalene (Laurdan; Sigma Aldrich). Briefly, for the fluorimetric assessment by the plate reader, cells were seeded out on a 96-well glass-bottom plate (Greiner, Stonehouse, UK). Cell membrane labeling with Laurdan was obtained by 5 to 10 min incubation with 5 μL of a solution in dimethyl sulfoxide (DMSO, Sigma Aldrich) and Phosphate Buffer Saline pH 7.4 (PBS, Sigma Aldrich), DMSO/PBS 1:3 v/v, at a final Laurdan concentration of 0.5 μM at room temperature; afterwards, cells were washed with PBS.

Fluorescence emission of Laurdan was exciting at 374 nm and recorded over its whole spectrum from 405 to 600 nm. The intensity of emission wavelengths at 440 ± 10 nm and 490 ± 10 nm was used to obtain the GP values. The experiments were done in triplicates employing 3 different cell batches; the fluorescence values observed were an average out of minimum 25 flashes. Additionally, for imaging purposes we added phenol-free L-15 cell media (Leibovitz, Lonza). Samples were prepared and imaged on an 8-well glass-bottom chamber #1.5 (Ibidi, Planegg/Martinsried, Germany).

### Generalized Polarization Index (GP)

Calculation of GP value was carried out as:

$$\begin{array}{l} GP=\frac{I_{440}-I_{490}}{I_{440}+I_{490}} \end{array}$$

where I_440_ and I_490_ correspond to the emission intensities of Laurdan at 440 and 490 nm, respectively, using 380 nm excitation wavelength. Values of GP vary from 1 to −1, where higher numbers reflect lower fluidity or higher lateral lipid order, whereas lower numbers indicate increasing fluidity. The GP measurements on live keratinocyte monolayers at different calcium concentrations (from [Ca2+] = 0.06 to 5 mM) were on one hand performed on a micro plate fluorescence reader (CLARIOstar, BMG LABTECH), with Laurdan fluorescence emission excited at 374 nm and recorded from 405 to 600 nm. The emission intensities at 440 and 490 ± 10 nm were used to obtain the GP values according to the above equation. On the other hand, confocal spectral imaging on live NHEKs at calcium concentrations [Ca2+] = 0.06 and 1.5 mM was performed on a Zeiss LSM 780 confocal microscope equipped with a 32-channel GaAsP detector array. Fluorescence of Laurdan was excited at 405 nm and detected between 415 and 691 nm. The images were then analyzed using a custom plug-in compatible with Fiji/ImageJ, as previously described 2 using a gamma variate fit of the spectra. A frequency histogram of the GP values was generated in Origin Pro (Oregon, United States), which disclosed two populations, one with high GP values representing the plasma membrane fraction and one with low GP values revealing the cytosolic fraction (e.g., from organelles). A fit of a double Gaussian distribution to the distribution allowed determining average GP values as well as standard deviations of both populations. In contrast, the plate reader measurements gave an average value over both populations. For both plate reader and spectral imaging *n* = 3 biological replicate were performed, while from each keratinocyte monolayer a minimum of 25 plate reader recordings or 15 confocal images (spectral images) were acquired and analyzed.

For the calculation of the GP index employing spectral imaging and rendering a GP map, we used a purposed-coded plug-in published elsewhere (Sezgin et al., 2015) and available at https://github.com/dwaithe/GP-plugin). Briefly, the plugin has in-built the GP general polarization formula and the discrete intensity values around the peak maxima (i.e., 440, and 490 nm) are obtained from a fitting to the spectrum obtained per pixel and extracting the values around these peak maxima from the nearest wavelength intervals. This methodology produces a spatial GP map representing the GP value for each pixel of the image. The outputs from these pixels are then exported into OriginPro (OriginLab, Oregon, United States) to produce the normalized frequency counts histograms per GP value. Every image pseudo-color representation has a corresponding custom look-up-table (LUT) matching the highest and lowest GP value obtained from the data.

## Results

### Assessment of Membrane Order in Live Human Epidermal Keratinocytes at a Single-Cell Level

**Figure 2:**
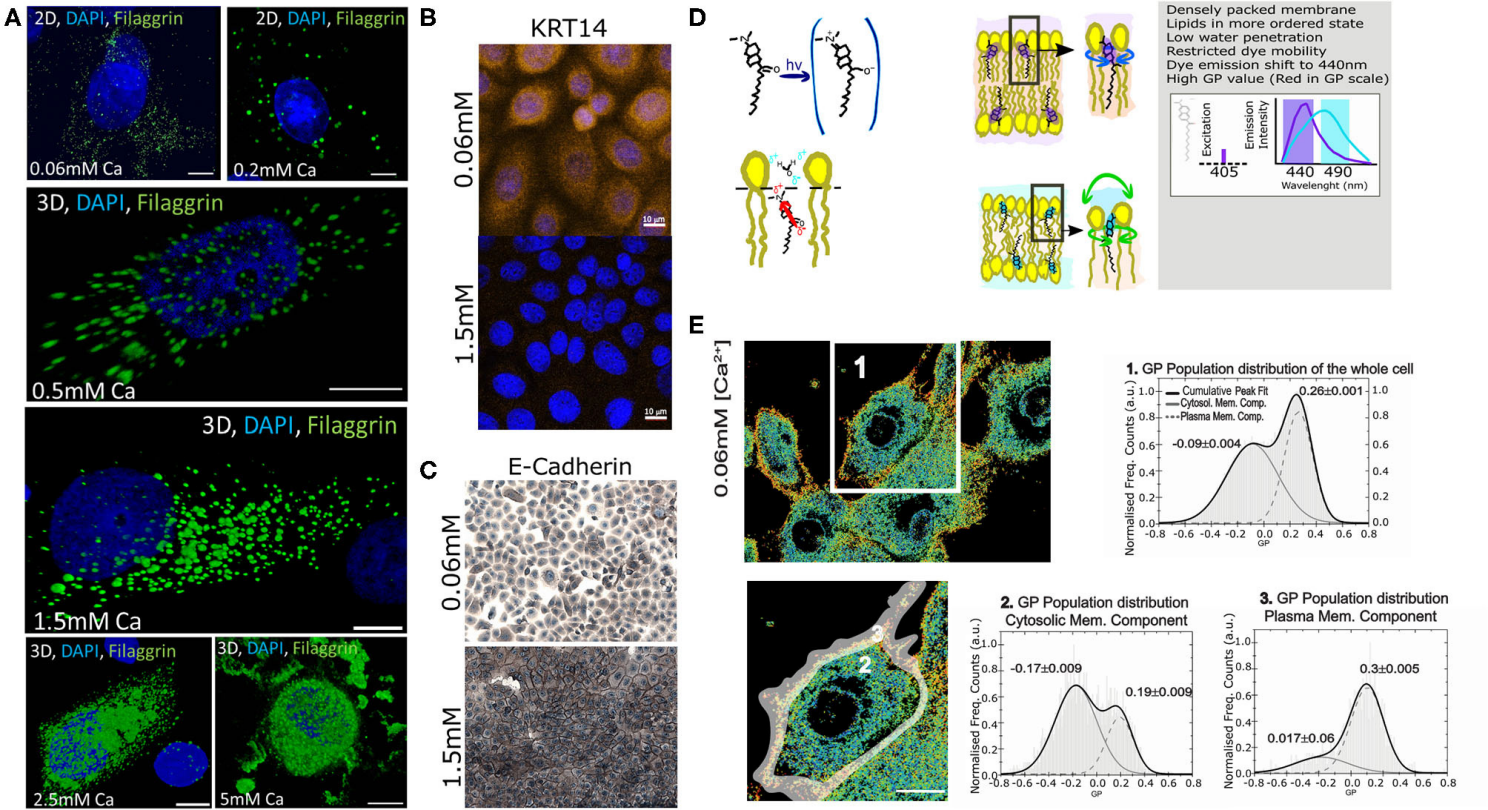
Assessment of the membrane order and stiffness in live normal human epithelial keratinocytes (NHEKs) by LAURDAN polarization sensing dye. **(A)** Detection of filaggrin-containing keratohyalin granules (KHGs) induced in primary normal human epidermal keratinocytes upon calcium switch by immunofluorescence ([Ca2+] = 5 mM to [Ca2+] = 2.5 mM); where Chromatin stained with DAPI is shown in Blue, and Filaggrin in green; scale bar 10 μm; **(B)** expression of keratin-14 and **(C)** E-cadherin staining to assess the formation of cellular junctions during calcium-induced differentiation; **(D)** A cartoon representing Laurdan chemical structure, its positioning within membranes and a description of its biophysical and fluorescence spectral properties. Loosely packed lipids and high water content promote unrestricted Laurdan mobility, resulting in dye emission shift representing fluid membrane state while dense lipid packing and lower water penetration restrict free movement of the dye, resulting in reflecting of shorter wavelengths; differences in LAURDAN emission reflects increased order of the lipids and is detected by a shift in emission wavelength (right panel). This results in an increase in the value of General Polarization (GP). **(E)** Pseudo-color scale represents the increase of a gradual shift from fluid (blue) to stiff (red) membrane. Representative GP images as determined from 2D scanning confocal spectral imaging on live undifferentiated NHEKS [Ca2+] = 0.06 mM; overview (upper panel, scale bar 5 μm) and magnified (lower panel, gray-shaded area: selection for plasma membrane part) of area marked by a white box. Pseudo-color scale: blue to red shift represent increase from low to high GP values. Frequency histograms of GP pixel values from areas of images marked by the respective number 1–3: (1) averaging over a whole cell; (2) cytosolic membranes fraction; and (3) plasma membrane fraction. The histograms were fit by a double-Gaussian (black line, with peak values, and their accuracy from the fit stated) to outline the plasma membrane (right peaks) and cytosolic (left peaks) fraction. Analysis from 3 different cell batches, and a minimum of 5 cells per batch; Statistical GP values obtained were −0.15+/−0.03 and 0.29+/−0.03 at cytosolic membranes and plasma membrane, respectively; the analysis of the whole cell yielded a double peak pit centered at a low GP −0.1+/−0.04 of and at a high GP of 0.21+/−0.05.

**Figure 3:**
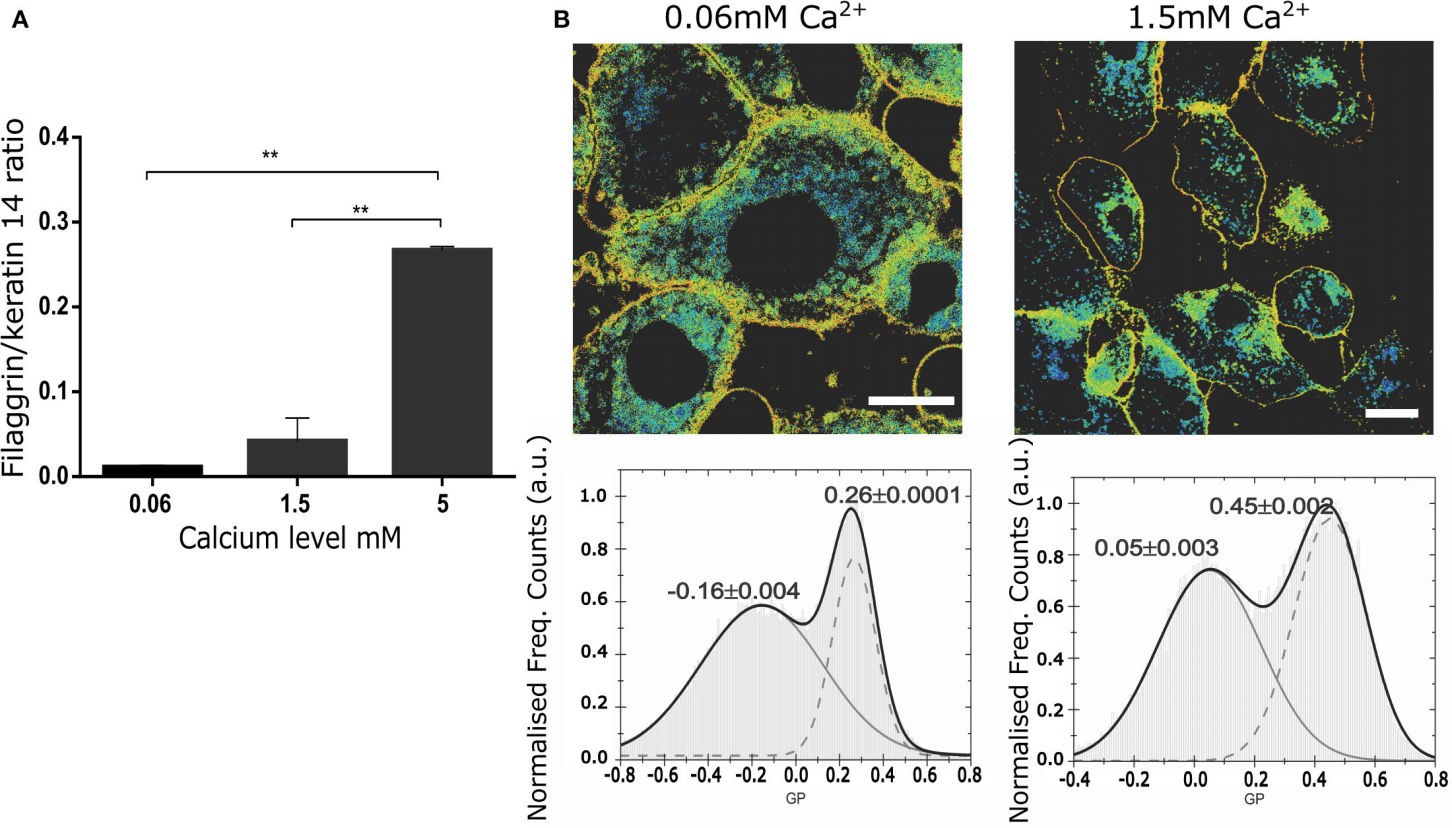
Changes in the membrane stiffness during *in vitro* keratinocyte differentiation assessed by LAURDAN live imaging at the level of a single cell. **(A)** Representation of the keratinocyte differentiation advancement by assessment of filaggrin/keratin 14 ratio in intracellular western blot assay in the cells used for LAURDAN assay; representative of 2 separate NHEK batches, assay carried out in duplicates; **(B)** GP images (upper panels) and histograms of GP pixel values (lower panels, from whole image) for increasing calcium levels ([Ca^2+^] = 0.06 mM (left), 1.5 mM (right)). The histograms were fit by a double-Gaussian (black line, with peak values and their accuracy from the fit stated) to outline the increase in GP and thus stiffness for the plasma membrane fraction (right peaks) and an indifferent change for the cytosolic fraction (left peaks). Representative example from 3 different cell batches, and a minimum of 5 cells per batch. Statistical membrane GP values obtained were 0.21+/−0.03 and 0.34+/−0.05 for [Ca^2+^] = 0.06 and 1.5 mM, respectively; scale bar 5 μm.

Previously, we extensively characterized the coordinated role of actin scaffolds and filaggrin granule formation during differentiation of normal human epidermal keratinocytes (NHEKs) (Gutowska-Owsiak et al., 2018). Specifically, we monitored filaggrin and actin at different calcium concentrations and described and quantified the distribution of these supramolecular assemblies in cornification. We noted that membrane molecular packing was following an interesting condensation pattern. Therefore, we decided to further investigate this and develop a fast, robust, and easy method to assess cornification development by quantifying lipid order in NHEKs. For this purpose, we used a “calcium switch” model of keratinocyte differentiation, whereby differentiation is induced by addition of calcium (Bikle et al., 2012), reflecting physiological changes observed during epidermal stratification (Menon et al., 1985). In this model a dramatic increase of filaggrin-positive granule accumulation in the cytosol and around the nucleus with higher calcium concentrations can be observed (Figure 2A). Furthermore, we observe expected changes in additional markers of keratinocyte differentiation (keratin 14 expression and formation of cellular junction), assessed by E-cadherin staining localization (Owens et al., 2000; Charest et al., 2009) as shown in Figures 2B,C, respectively. Combined, all these hallmark features span through the entire thickness of a live epidermis, i.e., represent early, intermediate and late differentiation stages of keratinocyte differentiation (keratin-14, adherens junction formation, and filaggrin, respectively) validate the assay as a suitable model to investigate keratinocyte differentiation. In order to characterize the lipid lateral packing properties in NHEK membranes, we directly labeled undifferentiated cells grown at low calcium level ([Ca2+] = 0.06 mM) with Laurdan. Next, we carried out spectral imaging by confocal microscopy to address changes in membrane stiffness reported by the quantification of the Laurdan general polarization (GP) index. This solvatochromic probe reports on the extent of water relaxation processes in its direct proximity in local molecular environment (Figure 2D). The additional advantage of Laurdan is that this dye only fluoresces when incorporated into a membrane environment, hence the GP index is unaffected by the minimal amount of Laurdan which is not associated to membranes. In cells, it is known that the overall internal organelle membrane composition differs from the plasma membrane (Bernardino de la Serna et al., 2016). Plasma membrane lipids have been reported to be more tightly packed and this is clearly demonstrated in Figure 2E GP pseudo-color map, where the plasma membrane display a redder color, indicative of a higher degree of condensation or more densely packed membranes in comparison to the intracellular membranes. Since our aim for this study was to develop a high throughput method to report on keratinocyte differentiation, we wanted to ensure that the overall quantification of the GP index would unambiguously report on the degree of cellular lipid lateral condensation, in a similar fashion to the quantification performed at the plasma membrane only. For this purpose, we compared the GP index population distribution and gaussian fit of a whole cell (Figure 2E-1) with the GP of the internal membranes (Figure 2E-2) and with the GP of the plasma membrane (Figure 2E-3). The distribution of GP values of the whole cell yielded two distinct gaussians: one with lower GP values, mostly distributed in the internal organelles and reflecting more fluid membranes (see Figure 2E in blue-green color; GP = −0.09); and the other with higher values, mainly localized at the plasma membrane, indicating higher degree of lateral packing (see Figure 2E in yellow-orange color; GP = 0.26). The values for the lower and higher GPs obtained from the whole cell, did not substantially differ from the lower GP values of the cytosolic membrane component and the higher of the plasma membrane component. This indicates that resolving the GP index distribution in cell membranes is sufficient to successfully characterize the lipid order heterogeneity.

### Laurdan General Polarization Index Increases During *in vitro* Keratinocyte Differentiation

Next, we tested whether confocal spectral imaging would show membrane order changes in live NHEKs during keratinocyte differentiation at a single cell level *in vitro*. Since a very steep calcium gradient in the epidermis (Menon et al., 1985, 1992; Elias et al., 1998, 2002; Ahn et al., 1999) is thought to be the main factor promoting differentiation and stratification *in vivo* (Hennings et al., 1980; Dale et al., 1983; Pillai et al., 1990), this model directly relates to skin physiology. Initially, we validated our assay by simultaneous determination of the differentiation progression in the cells using proteomics (end point assessment). Here, we used the ratio between the expression of a late differentiation marker (filaggrin) and keratin 14, which is predominantly expressed by undifferentiated keratinocytes of lower epidermal layers, including in the basal layer characterized by high proliferation rates (Figure 3A). The increase of the filaggrin/keratin 14 ratio indicates keratinocytes advancing in their differentiation process.

Next, using spectral imaging we investigated whether quantifying the GP index in live NHEKs in our validated differentiation model would yield differences that could be confidently used to assess cornification by the means of membrane order quantification. Specifically, we exposed NHEKs to increasing calcium level for 24 h, labeled them with Laurdan and determined GP imaging maps and GP index population distributions. The GP maps in Figure 3B confirmed the overall cellular membrane state, showing a decrease in fluid membranes overall and an increase in more ordered membranes. Moreover, the histograms showed a clear and significant response of the membrane packing readout in both the low and high GP value with increased calcium concentration. For instance, from 0.06 mM to 1.5 mM Ca^2+^ the whole population distribution is shifted toward higher GP values: from −0.06 to 0.05 and from 0.26 to 0.45 for the low and high GP, respectively. Interestingly, the highest calcium concentration (5 mM) shows complete disruption of the cells and their membranes. Plasma membrane stiffness and thus GP values increased with calcium concentration throughout the tested range (0.06–5 mM). Given this, we concluded that GP values hold as a robust readout for determination of differentiation stages of keratinocytes at the single live-cell level.

### High Throughput GP Index Quantification Assay to Determine Progression of Differentiation in Live Keratinocytes

**Figure 4:**
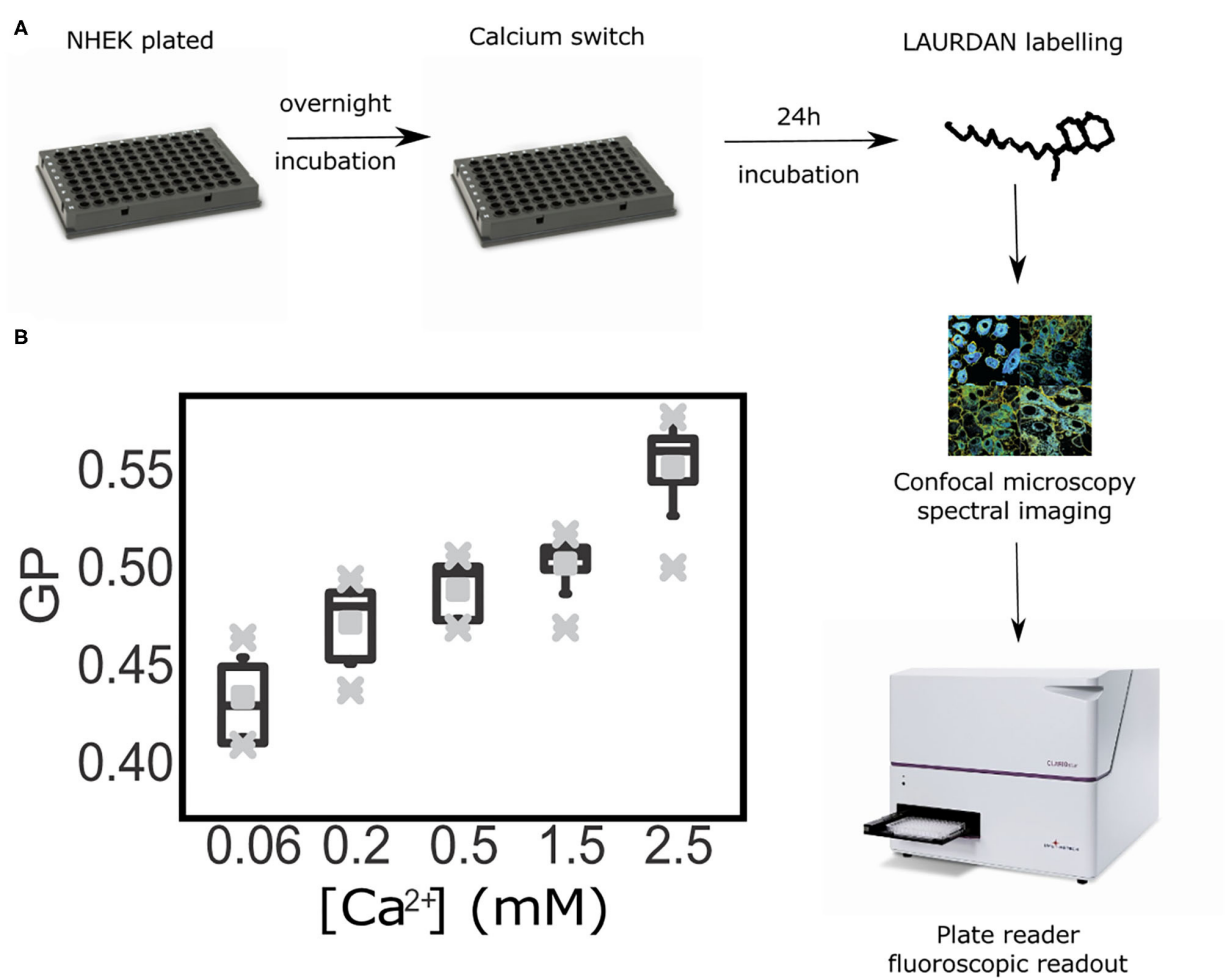
High throughput assessment of Laurdan General Polarization (GP) in living keratinocytes at the population level by a fluorescent plate readout. **(A)** Workflow diagram: primary keratinocytes were seeded and calcium-switched in a range of calcium concentrations ([Ca2+] = 0.06 mM to [Ca2+] = 2.5 mM) at confluence. Twenty four hour after cells were LAURDAN-stained live and GP values were obtained by confocal immunofluorescence readout and fluorescence spectroscopy on a plate reader. **(B)** Whiskers-box plot of the distribution of GP values, with average (middle vertical black lines), the standard deviations (error bars), and the outlier ranges (gray crosses).

Once we demonstrated that the GP index is a suitable reporter of cell membrane order without the need to obtain values exclusively from the plasma membrane, we progressed further in our aim of developing an inexpensive and robust methodology to asses keratinocyte differentiation with a high throughput readout and analysis. For this, we tested whether calculating the GP index at the cell-ensemble level, rather than at the single-cell, would yield corresponding results. Hence, we reproduced the previous experimental set up, but employing a plate reader instead of a confocal microscope. This assay provided average GP values from whole cell populations within sub-second time frames. In these experiments we determined GP values representative of all cell membranes, i.e., calculating the average over both the cytoplasmic and cytosolic membrane fractions. This new approach allowed us to test multiple calcium concentrations faster and in a larger field of view; ultimately allowing access to a more robust statistical analysis. A caveat to this approach is the fact that the readout is an average of the fluorescence spectra detected from an ensemble of keratinocytes within a cell monolayer. To further test whether this could be a set-back to our method, we ran the samples in parallel in a plate under a confocal microscope and in a plate reader (Figure 4A). Notably, using the same cell populations as imaged on the confocal microscope (the plate format fitted both the microscopy setup and the fluorescent reader) we could still observe a gradual increase of the Laurdan GP values along with the progression of keratinocyte differentiation in our calcium switch model (Figure 4B). The low error bars along with the short measurement times highlight the plate reader assay as a robust high-throughput readout to assess keratinocyte differentiation.

## Discussion

Epidermis consists of multiple layers of keratinocytes which represent different stages of progression of terminal differentiation. Successful and complete differentiation is critical for the functional characteristics of the skin, and affects their permeability, antimicrobial, and mechanical properties. While the cells gradually progress, their characteristics change dramatically; this results in differential cell reactivity and outcomes in functionally distinct keratinocyte populations. To date there are no specific tools available to trace the progression of keratinocyte differentiation *in vitro* in real-time or to precisely determine differentiation advancement stage of these cells while preserving cell viability; at neither single-cell nor population level. Such an assessment modality would be beneficial, however, as it would enable the determination of the dependence between the cell reactivity and functional outcomes of the advancement in their differentiation program. Furthermore, a plate reader format could provide an efficient tool for scaling up into a high-throughput assay. Similarly, given that several skin diseases have been found to show a degree of delayed or abnormal epidermal differentiation and resulting insufficiency in the skin barrier function (Kubo et al., 2012; Eyerich et al., 2018), there is an unmet need for robust and validated techniques which could be used to propose new diagnostic and therapeutic options. Our study provides an inexpensive and effective methodology to assess keratinocyte differentiation and has implications to both basic and applied research, e.g., in drug testing. However, it is important to note that while our assay may be used to determine the GP value and make assumptions with regards to the membrane stiffness across a spectrum of calcium concentrations, the cells grown at calcium levels of 5 mM or above may no longer be viable, likely due to differentiation-dependent cell death and not suitable for downstream analysis. Another important aspect to consider is the fact the Laurdan GP is not an absolute, but relative value; the values are indicative of a process of differentiation, where clearly the order in the cellular membranes increases upon higher calcium levels. As shown, absolute value differences in the analyses can be attributed to (i) the lower sensitivity of the plate reader compared to the microscope detectors; (ii) the excitation power and wavelength (in a LED vs. laser-based for the microscope); and (iii) the difference in the cellular organization (ensemble of cells, where cell-cell attachments influence the membrane fluidity, vs. single-cell visualization under the microscope).

Lipid membrane remodeling and resulting changes in the polarity can be employed to measure the degree of membrane condensation by utilizing fluorescent lipophilic probes such as Laurdan, where emission spectra and solvent-polarity dependence report on lipid lateral packing. The readout of the Laurdan fluorescence and GP index can be used to provide information regarding membrane heterogeneity, which allows for the estimation of the membrane stiffness and fluidity. Furthermore, lack of the apparent Laurdan toxicity and the sub-second readout time of the plate reader allowed us to study changes in keratinocyte stiffness and progression of differentiation over time. It is important to note that the GP index is not an absolute measure of fluidity, but an indirect and relative estimate. Therefore, GP values may differ from study to study. For instance, very precise measurements at room temperature of GP in gradually laterally compressed monolayers of the di-saturated lipid DPPC (1,2-dipalmitoyl-sn-glycero-3-phosphocholine), a lipid with a transition temperature of 41°C, lead to GP values between 0.25 and 0.4 (Brewer et al., 2010) at lateral pressures, similar to those experienced in a lipid bilayer in the liquid order phase (de la Serna et al., 2009; Bernardino de la Serna et al., 2013). However, when DPPC is compressed further to reach a gel state (e.g., a phase that has only been reported in skin membranes or model membranes) the GP value still reaches values below 0.5. Therefore, the values reported here for highly densely packed membranes of are in good agreement with the increase of the membrane order (de la Serna et al., 2009; Gutowska-Owsiak et al., 2018).

In this study we demonstrated that Laurdan general polarization (GP) index can be used as an effective tool to determine keratinocyte differentiation advancement and progression in live keratinocytes at both single-cell and population level, within a cell monolayer and with an option for high-throughput assessment. Laurdan has been previously used in experiments with porcine skin to determine penetration of liposomes (Carrer et al., 2008). Assessment of Laurdan emission in these studies showed a decrease of the GP value with the increased skin depth, from *stratum corneum* through to the dermis, including a progressive decrease at the level of the epidermis; this is in line with data presented here. While we utilized Laurdan to assess differentiation process in epidermal keratinocytes specifically, we anticipate that this methodology may be also potentially successfully used with other cell types, which undergo lipid remodeling during differentiation or maturation (Reynier et al., 1991; Wong et al., 2018).

In summary, we designed and validated a Laurdan-based assay which enables for a precise determination of differentiation advancement stage in living keratinocytes, by the assessment of changes in membrane stiffness. Due to the lack of apparent cytotoxicity, this *in vitro* assay may also be used to trace differentiation over time, either at the level of a single cell or scaled up for high throughput measurements.

## Data Availability Statement

The raw data supporting the conclusions of this article will be made available by the authors, without undue reservation.

## Author Contributions

DG-O and JBS designed and performed the experiments, carried out the data processing and analysis, discussed, prepared, and wrote the manuscript. EIP carried out experiments. CE and GO supervised the work, helped with analysis, discussed, prepared, and wrote the manuscript. All authors contributed to the article and approved the submitted version.

## Conflict of Interest

The authors declare that the research was conducted in the absence of any commercial or financial relationships that could be construed as a potential conflict of interest.

## Supplementary Material

Supplementary Figure 1 Validation of anti-filaggrin antibodies used in the study. Western blot assessment of the antibody specificity for FLG01 and 15C10 antibodies used in the study. **(A)** Blotting against recombinant human FLG protein; **(B)** Blotting against epidermal lysates.
