# Supplementary figures and images for: Addressing Differentiation in Live Human Keratinocytes by Assessment of Membrane Packing Order
Source: Front Cell Dev Biol. 2020 Oct 21;8:573230. doi: 10.3389/fcell.2020.573230 (PMC7609878; doi:10.3389/fcell.2020.573230)

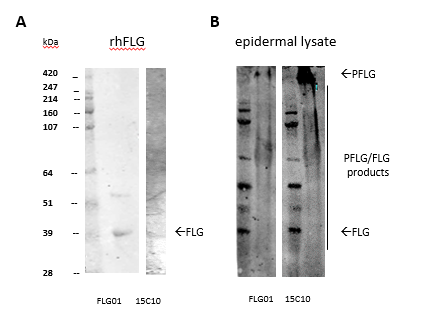

Supplement: Supplementary Figure 1 — Validation of anti-filaggrin antibodies used in the study. Western blot assessment of the antibody specificity for FLG01 and 15C10 antibodies used in the study. (A) Blotting against recombinant human FLG protein; (B) Blotting against epidermal lysates. [file Image_1.PNG]
